# Immunomodulatory properties of morphine and the hypothesised role of long-term opioid use in the immunopathogenesis of tuberculosis

**DOI:** 10.3389/fimmu.2023.1265511

**Published:** 2023-10-23

**Authors:** Vipula R. Bataduwaarachchi, SMN Hansanie, Neesha Rockwood, Leon Gerard D'Cruz

**Affiliations:** ^1^ Department of Pharmacology, Faculty of Medicine, University of Colombo, Colombo, Sri Lanka; ^2^ Research and Innovation Department, Portsmouth Hospitals University NHS Trust, Portsmouth, United Kingdom; ^3^ Department of Microbiology, Faculty of Medicine, University of Colombo, Colombo, Sri Lanka; ^4^ Department of Infectious Diseases, Imperial College London, London, United Kingdom; ^5^ School of Pharmacy and Biomedical Sciences, University of Portsmouth, Portsmouth, United Kingdom

**Keywords:** immunopathogenesis of tuberculosis, morphine, opioids, opioid receptors, toll-like receptors, immunosuppression

## Abstract

Epidemiological studies have shown high tuberculosis (TB) prevalence among chronic opioid users. Opioid receptors are found on multiple immune cells and immunomodulatory properties of opioids could be a contributory factor for ensuing immunosuppression and development or reactivation of TB. Toll-like receptors (TLR) mediate an immune response against microbial pathogens, including *Mycobacterium tuberculosis*. Mycobacterial antigens and opioids co-stimulate TLRs 2/4/9 in immune cells, with resulting receptor cross-talk via multiple cytosolic secondary messengers, leading to significant immunomodulatory downstream effects. Blockade of specific immune pathways involved in the host defence against TB by morphine may play a critical role in causing tuberculosis among chronic morphine users despite multiple confounding factors such as socioeconomic deprivation, Human immunodeficiency virus co-infection and malnutrition. In this review, we map out immune pathways involved when immune cells are co-stimulated with mycobacterial antigens and morphine to explore a potential immunopathological basis for TB amongst long-term opioid users.

## Introduction

Tuberculosis (TB) remains a longstanding global health challenge, claiming 1.5 million lives in 2020. Two-thirds of new cases come from the eight highest-burdened nations, with six of them in South and Southeast Asia. (1)population is latently infected with TB. The risk of reactivation of latent *Mycobacterium tuberculosis* (MTB) is rising due to international travel, migration, immunosuppressive co-morbidities, and medication use, affecting both developed and developing countries. (2) In order to achieve the World Health Organization goal of reducing 90% of new TB cases by 2035, it is vital to better understand the key causes of reactivation of latent TB. Multiple risk factors for TB are clustered in different subpopulations. (3–5) Epidemiological data have shown that long-term opioid users are more susceptible to TB than the rest. (6) As per a comprehensive community-based case-control study, higher TB risk was independently associated with tobacco smoking, drug use (especially injectable drugs OR = 5.67; 95%CI: 2.68, 11.98), homelessness and area-level deprivation. The strongest risk factor among the intermediate social determinants was misuse of class A injectable drugs (e.g., Ecstasy, Cocaine, Crack Cocaine, Heroin), with five times higher TB odds (OR = 5.67; 95%CI = 2·68, 11.98) compared to those who never misused class A drugs (adjusted for age, sex, BCG vaccination status and long stays in high TB area). (7) A multivariate analysis has shown that drug use was associated with smear-positive TB (OR 2.2, CI 311 – 401, p<0.001). (8) Even accounting for genetic, environmental, socio-economic, and culture-related risk factors, raises the possibility of an underlying immunopathological basis leading to immune suppression. (6, 9–11) TB is often the most common opportunistic infection in endemic areas. (11) This highlights the importance of exploring independent pathophysiological mechanisms causing immune impairment in chronic drug use separately in different infections despite the multiple confounding factors causing generalised immune suppression.

The *World Drug Report* of 2021 has reported that around 275 million people used illicit substances in 2020 globally, highlighting another growing health challenge. Opioids are the most heavily used substance. Over 75% of substance users live in developing countries, where the prevalence of TB is also highest. (12) Long-term use takes different forms, including misuse of prescription opioids, habitual use without dependence, and increased use as a long-term analgesic. (13) A recent review published in the *Lancet* has highlighted the TB burden among vulnerable groups worldwide with variable prevalence. (14) **Table 1** further summarises studies assessing a link between drug use and TB. Except for one study, others point towards a strong link between drug use and TB risk. As multiple confounders co-exist in these vulnerable populations, causality cannot be independently assessed. Epidemiological data assessing TB infection among drug users taking it as an independent variable is a future need. More specific details of the nature of drug use and the immune status will further help fill the missing data gap.

**Table 1:** Summary of the epidemiological data assessing tuberculosis among drug users.

Study population	Type of study/analysis	Detection rate of TB	Confounding risk factor assessment	Ref
Ukraine	Cross-sectional study	N=680,760 records (68% PWID); 20% of presumptive TB cases were detected among PWID	–	(15)
Global	Systematic Review	N= The overall denominator not quantified.; The prevalence of TB is higher in prison populations than in the general population, mainly because of the criminalisation of drug use and the detention of PWID or use drugs	–	(16)
Vietnam	Cross-sectional survey	N=885 (PWID); TB prevalence was 2.3% (95% confidence interval [CI], 1.0–4.5) and 2.1% (95% CI, 0.8–4.2) among HIV-positive and HIV-negative people, respectively.	Multivariable exact logistic regression analysis was used with TB as the dependent variable. HIV infection was not associated with TB but rather with an accumulation of individual risk factors.	(17)
Abidjan, Ivory Coast	cross-sectional prevalence estimation survey	N=532 (PWID) (with an Xpert MTB/RIF^®^ test result), 52 had pulmonary TB, a prevalence of 9.8%, 95% CI [7.5%-12.7%]. Among them, 17.3% had rifampicin-resistant TB. The drug most commonly consumed was heroin (n = 530; 99.6%)	Multivariable analysis Factors significantly associated with TB infection: being co-infected with HIV	(18)
Tanzania	Prospective cohort study	N=901 records (53.5% PWID); TB disease and TB infection prevalence was 2.6% and 54%, respectively.	Adjusted logistic regression model adjusting for risk factors, the risk of TB infection was reduced in PWID. (OR 0.67, 95% CI 0.49–0.90)	(19)

TB, Tuberculosis; PWID, People with injectable drugs; MTB, Mycobacterial tuberculosis; HIV, Human immunodeficiency virus; OR, Odds ratio; CI, Confidence interval.

Immunomodulatory properties of opioids vary in the presence of different microbial antigens. Detailed characterisation of immune pathways affected by opioids in the presence of specific microbial pathogens is essential to characterise mechanisms of immunopathogenesis further. (20, 21) Due to the widespread testing of morphine in multiple previous pre-clinical studies (22), this review collates the immunomodulatory properties of morphine, and it suggests a potential role for opioids during chronic use in the immune pathogenesis of TB.

## Opioid receptors in the immune system

Opioids act on their receptor (OR), a G protein-coupled receptor (GPCR). They are categorised into two distinct groups: classical OR, which includes *morphine* (mu) MOR, *ketocyclazocine* (kappa) KOR, and *vas deferens* (delta) DOR, and non-classical OR, which includes nociceptin Orphanin FQ peptide receptor (NOR). (23, 24) ORs are located in multiple immune cells, including macrophages and lymphocytes, and are widely distributed in the human body. Both endogenous opioid peptides and exogenous synthetic opioids with different molecular properties act on the same receptors, giving rise to variable downstream effects. (21, 25)

Chronic morphine administration is known to cause impairment of both innate and adaptive arms of the immune response. (26) The concept of direct and indirect morphine action was on the immune system first introduced through work in preclinical studies, which indicated that the MOR mediates morphine-induced immunosuppression and that although some functions are amplified in the presence of cortisol or sympathetic activation. (27) The activation of KOR has also been shown to reduce antibody production, inhibit phagocytic cell activity, inhibit T cell development and alter the production of various pro-inflammatory cytokines, chemokines, and the receptors for these mediators. (28) *In vitro* experiments have shown that the DOR agonist KNT-127 causes immune suppression in rat models with colitis. (29) It has further shown the functional differentiation of OR subtypes located on the immune cells responding differently to endogenous and exogenous opioids. The novel M3OR subtype has been characterised as an opioid peptide-insensitive and opiate alkaloid-selective GPCR that is functionally linked to constitutive nitric oxide synthase activation. Opioid peptides stimulate granulocyte and immunocyte activation and chemotaxis via the activation of a novel leukocyte D2OR subtype. However, opiate alkaloid M3OR agonists inhibit these same cellular activities. (30)

Chemokine receptors (CCR), which mediate chemokine response, belong to the same class of GPCR as OR and possibly share a common evolutionary origin. The evidence suggests that these receptors cross-desensitise each other, whereas morphine that binds to OR can block CCR signalling and vice versa. (31, 32) The mechanisms underlying heterogenous desensitisation could be the formation of receptor heterodimers and protein kinase C-mediated phosphorylation of Serine, Threonine and Tyrosine moieties. Heterogenous desensitisation may be one mechanism of immune suppression by opioids with high doses and long-term exposure. (33, 34) Bivalent molecules can be tested to modify the complex and its cellular effects. Different immune effects mediated by OR in the presence of mycobacterial antigens and morphine are discussed in the subsequent sections. Bivalent molecules that selectively modulate the OR-CCR complex have therapeutic potential, such as VZMC013, which targets the MOR-CCR5 heterodimer to inhibit opioid-exacerbated HIV1 entry into the immune cells. Similar molecules may have applications in managing opioid-induced immune suppression, including the potential TB risk (35).

## Modulation of the immune cells and mediators by morphine

A focused literature search in Google Scholar, PubMed and Medline was carried out to extract the studies conducted to find the effects of ‘morphine’ on the ‘immune system’. **Tables 2**, **3** comprehensively summarise the immune cells and mediators influenced by morphine administration. Morphine suppresses multiple immune cells, including macrophages, which play a crucial role in the immunopathogenesis of TB. The consequences of morphine exposure extend to inhibiting chemotaxis and multiple cellular functions in macrophages, including respiratory burst activity, phagocytosis, and colony formation (**Table 2**).

**Table 2:** Effects of morphine on the immune cells.

Cell type	Treatment mode of opioids	Type of Study	Effects observed	References
NK cells		*In vivo murine*	Suppressed activity in the spleen via a neuron-mediated mechanism	(36–38)
	A	*In vivo murine*	Suppressed via adrenergic and sympathetic neurotransmitters, glucocorticoid, dopaminergic, and peptide Y signalling	(39–43)
	A	*In vivo murine*	PAG administration – suppressed activity	(44, 45)
	A	*In vivo human*	Intrathecal administration – suppressed activity	(46)
	DD	*In vivo pigs*	Increased cytotoxicity	(47)
		*In vitro*	Suppression cytotoxicity via MOR and KOR agonists but not DOR agonists	(48)
DC		*Ex vivo murine*	Reduced activity and antimicrobial proteins via TLR2 and NLR2 signalling mechanisms	(49)
Splenic/thymic/LN lymphocytes	SR	*In vivo murine*	Induced atrophy	(50, 51)
	SR	*In vivo murine*	Altered CD4/CD8 ratio	(52)
	SR	*In vivo Rhesus monkey*	Altered CD4/CD8 ratio	(53)
	SR	*In vivo murine*	Reduced B cells (Ig M+/Ig D-), CD4 and CD 8 cells (naïve and effector memory depleted)	(54)
		*In vitro murine*	Reduced NFAT binding to DNA and decreased IL2 production	(55)
		*In vivo murine*	Reduced cellularity and induced Fas	(56)
Mononuclear cells, including PBMC	SR	*In vivo Rhesus monkey*	Reduced IL2r expression	(57)
		*In vitro human*	Induced apoptosis via Fas	(56)
		*In vitro human*	Induced apoptosis via NO	(58, 59)
		*In vitro human/murine*	Th2 switch with increased IL4 and IL5 and decreased IL2 and IFN γ	(60)
Macrophages		*Ex vivo human*	Inhibited chemokine-mediated chemotaxis	(61)
		*In vitro & in vivo*	Reduced phagocytosis due to reduced SO anion directly via MOR	(62–66)
	C	*In vitro & in vivo*	Inhibited phagocytosis by MOR and D2OP in a dose-dependent manner by inhibiting actin polymerisation via the inhibition of Rac1-GTPase and p38 MAPK	(51)
		*Ex vivo murine*	Reduced respiratory burst activity (morphine-stimulated NO release mediated by an M3OR subtype expressed on the surface of monocytes, in contrast to fentanyl)	(67, 68)
		*In vitro murine*	Inhibited macrophage colonies	(69)
Leucocytes		*In vivo murine*	Reduced sticking and rolling along the blood vessels	(70)
T lymphocytes	A	*In vitro murine*	Reduced response to ConA via a centrally acting mechanism	(71, 72)
		*In vivo murine*	Recused calcium reflux in CD4+ via a glucocorticoid-mediated mechanism	(73)
B lymphocytes		*In vitro murine*	Reduced proliferation stimulated by IL4 and anti-IgM via a centrally acting mechanism	(74–76)
		*In vivo murine*	Inhibition of calcium mobilisation is an early event in opiate‐induced immune suppression.	(73)
		*Ex vivo human*	IL8-mediated chemotaxis	(61)
		*Ex vivo human*	Reduced SO production	(77)

***A***, acute; ***DD***, dose-dependent; ***SR***, slow release; ***C***, chronic; Con A, Concanavalin A; DNA, Deoxyribonucleic acid; DOR, δ-opioid receptor; GTPase, guanosine triphosphate ase; IFN-γ, Interferon-gamma; IL, Interleukins; KOR, κ-opioid receptor; MAPK, Mitogen-activated protein kinase; MOR, μ-opioid receptor; NFAT, Nuclear factor of activated T cells; NLR, Nucleotide oligomerisation domain like receptor; NO, Nitrous oxide; PAG, Periaqueductal grey; SO, Sulphur oxide; TLR, Toll-like receptor.

**Table 3:** Morphine effects on the chemical mediators.

Component of the immune system	Mediators	Type of Study	Effects observed	References
**Macrophages**	IL1β, IL6, IFN γ	*Ex vivo murine*	Reduced levels	(78)
	IL1β, IL 6, TNFα	*Ex vivo murine*	Reduced levels (KOR)	(79)
	NO, IL4, MMP9, arginase 1	*Ex vivo murine*	Reduced levels	(80–82)
	IL10	In vivo murine* (In both WT/RelB-/-)	Reduced levels	(83, 84)
	IL10	*Ex vivo human*	Increased levels	(85)
	IL12	In vivo murine* (Only in WT, not in RelB-/-)	Reduced levels	(83, 84)
	IL12, TNF α	*In vivo murine*	Increased levels	(86)
	IL10	*In vivo murine*	Unchanged	(86)
	IL6, TNF α	*In vitro murine*	Increased levels with low dose Reduced levels with high dose	(87)
**DC as well**	IL23	*Murine*	Reduced levels	(56, 68, 69)
**PBMCs**	Reactive oxygen species, O2−, H2O2	*Ex vivo human*	Reduced levels	(88)
	IFN γ, TNF α	*Ex vivo human*	Reduced levels	(88, 89)
	CCL2, CCL5, CXCL10	*Ex vivo human*	Increased levels	(90)
	TGF β1	*Ex vivo human*	Increased levels	(91)
**T lymphocytes**	IL17	*In vivo murine*	Reduced levels	(92)
	IL4	*In vivo murine*	Reduced levels	(93)
	IL 2	*Murine*	Reduced levels	(89, 92)
	TNF α, IL1β, IL4, IFN γ	*In vivo murine*	Reduced levels (via MOR)	(94)
**Neutrophils**	MPO	*In vitro*	Weak reversible inhibitor	(95)
**Astrocytes**	CCL2, CCL4, CXCL1	*In vitro human*	Reduced levels	(96)
	CCL5, CCL12	*In vitro murine*	Reduced levels	(97)
	CXCL10	*In vitro human*	Increased levels	(98)
**Intestinal epithelial cell line**	IL8	*In vitro human*	Reduced levels (via KOR)	(99)
	IL8	*In vitro human*	Increased levels (MOR)	(100)

*Same study, related.

CCL, Chemokine ligand; CXCL, C-X-C motif chemokine ligand; H_2_O_2_, Hydrogen peroxide; IFN γ, Interferon gamma; IL, Interleukins; KOR, κ-opioid receptor; MMP, Matrix metallopeptidase; MOR, μ-opioid receptor; MPO, Myeloperoxidase; NO, Nitrous oxide; RelB, v-rel reticuloendotheliosis viral oncogene homolog B; TGF, Transferrin growth factor; TNF, Tumour necrosis factor; WT, Wild type.

**Figure 1:**
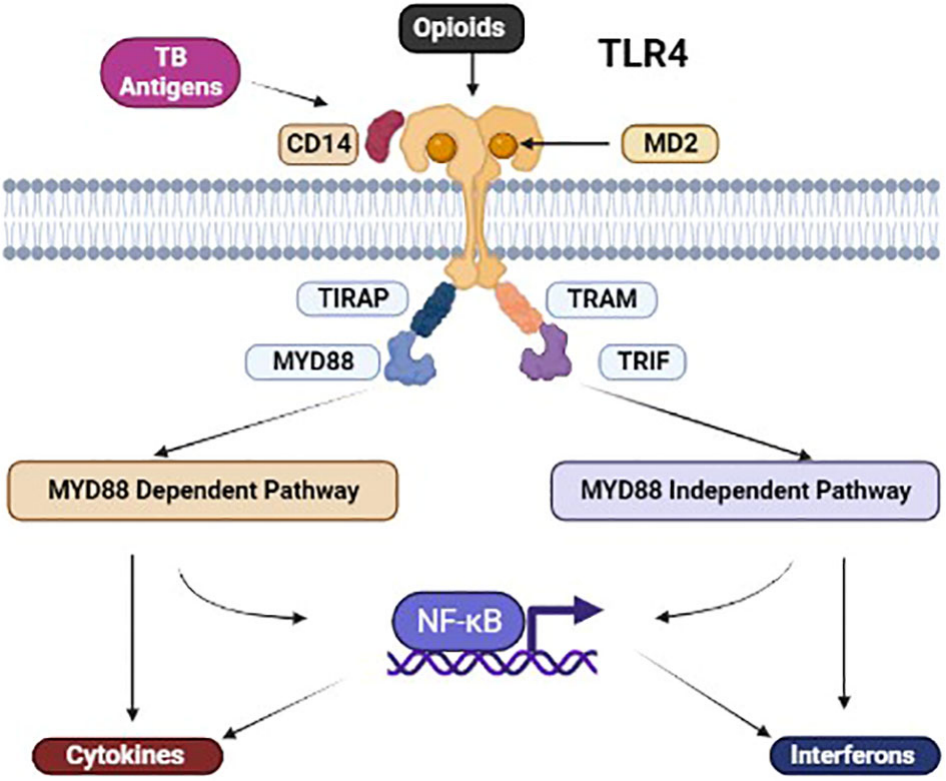
Illustration of the co-binding of M. tuberculosis (MTB) antigens and opioids with TLR4. Illustration of the co-binding of MTB antigens and opioids with TLR4. Co-stimulation of the TLR4 leads to MyD88-dependent and independent activation, leading to NFkB-dependent and independent mechanisms affecting chemical mediators downstream. [TLR, toll-like receptor; NF-κB, Nuclear factor-κB; TIRAP, Toll-interleukin-1 Receptor (TIR) domain-containing adaptor protein; TRIF, TIR-domain-containing adaptor-inducing interferon-β; TRAM, TRIF-related adaptor molecule].

Suppression of the critical immune mediators such as tumour necrosis factor (TNF α), interferon (IFN γ), and nitrous oxide (NO) produced by macrophages was also reported in multiple studies (**Table 3**). **Figure 1** illustrates the immune pathways affected during the co-stimulation of macrophages by mycobacterial antigens and morphine. Suppression of the neutral killer (NK) cell by morphine is modulated via direct and centralised mechanisms. Lymphocytes are inhibited by multiple means, including reduced cytotoxicity and altered CD4/CD8 cell ratios.

## The role of TLR during the interaction with mycobacterial antigens and morphine

TLRs are found in various immune cells and play a critical role in recognising molecular patterns of pathogens to trigger the immune system. Multiple TLRs, including TLR2, TLR4, TLR8, and TLR9, interact with various mycobacterial antigens. Morphine interacts with TLR2, TLR4, and TLR9, and co-stimulation by morphine and mycobacterial antigens may lead to complex immune effects downstream (49, 101–105).

**Figure 2:**
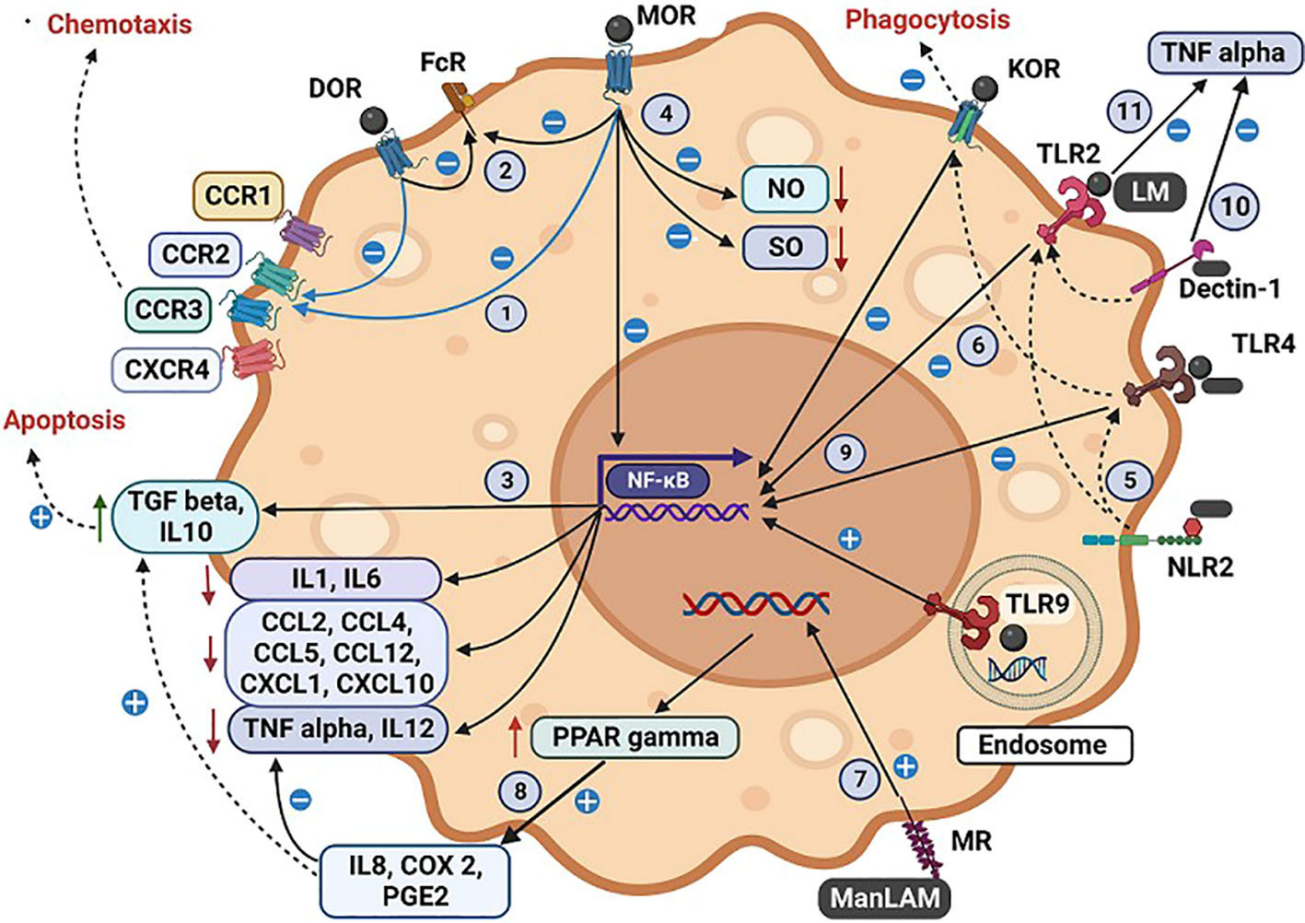
Illustration of the interactions by tuberculous antigens and morphine with the cell surface and intracellular receptors of a macrophage. The interaction between tuberculous antigens and morphine within macrophages. Morphine (M) and Mycobacterial antigens engage with TLR 2/4/9, while Mycobacterial antigens interact with NLR, MR, and Dectin-1. M enhances Mycobacterial antigen virulence by inhibiting TLR 2/4 and OR. Morphine influences various macrophage functions: (1) Desensitizing multiple CCRs via MOR and DOR. (2) Inhibiting FcR-mediated apoptosis via MOR, DOR, and KOR. (3) Enhancing TGF-induced apoptosis. (4) Impairing NO and SO synthesis, respiratory burst activity, and bacilli killing. NLR2 augments TLR 2/4 actions through cross-talk. (6) M inhibits NFκB-mediated cytokine and chemokine synthesis via OR-TLR cross-talk. TLR9 elicits a proinflammatory response with Mycobacterial antigens and M, while TLR2/4 induces an anti-inflammatory response. (7) MR, present in AM, regulates protective macrophage responses. MTB or ManLAM upregulates PPARγ via MR, increasing IL8, COX2, and PGE2. ManLAM generates an anti-inflammatory response, inhibiting proinflammatory TNF and IL12 while inducing immunosuppressive IL10 and TGF β. ManLAM signalling via TLR2 and TLR4 triggers chemokine secretion in monocytes, with M exerting inhibitory effects via TLR 2/4 (10). Dectin-1, in combination with TLR2, induces TNF production in macrophages, particularly in attenuated MTB strains (11). LM blocks TLR2-induced TNF biosynthesis, permitting MTB to evade the host immune response. Antigen-specific variations are noted, with M-induced immune suppression amplifying antigen virulence mechanisms. (*A more detailed version of this figure legend is provided in the **Online Data Supplement***).

Multiple mycobacterial antigens interact with immune cells via TLR-dependent and independent mechanisms. **Figure 1** illustrates the co-binding of mycobacterial antigen and opioids with the TLR4 and its adaptor proteins. Rapidly growing, non-pathogenic mycobacteria containing AraLAM in their cell walls activate CD14 cells expressing TLR2 and macrophages. In contrast, slow-growing pathogenic MTB containing ManLAM has shown a relative inability to activate macrophages independent manner, potentially contributing to their virulence (**Figure 2**). However, other soluble and cell wall-associated mycobacterial antigens distinct from LAM can mediate immune cell activation via TLR. For example, a soluble heat-stable and protease-resistant factor mediates TLR2-dependent activation of immune cells, whereas a heat-sensitive cell-associated mycobacterial factor mediates TLR4-dependent activation of them. (106–112) Interestingly, induction of adaptive T cell response in TB does not require TLR2/4/9. In TLR2/4/9-deficient mice, mycobacterial replication is controlled by TLR-independent mechanisms to induce an adaptive T-cell response (113).

**Figure 3:**
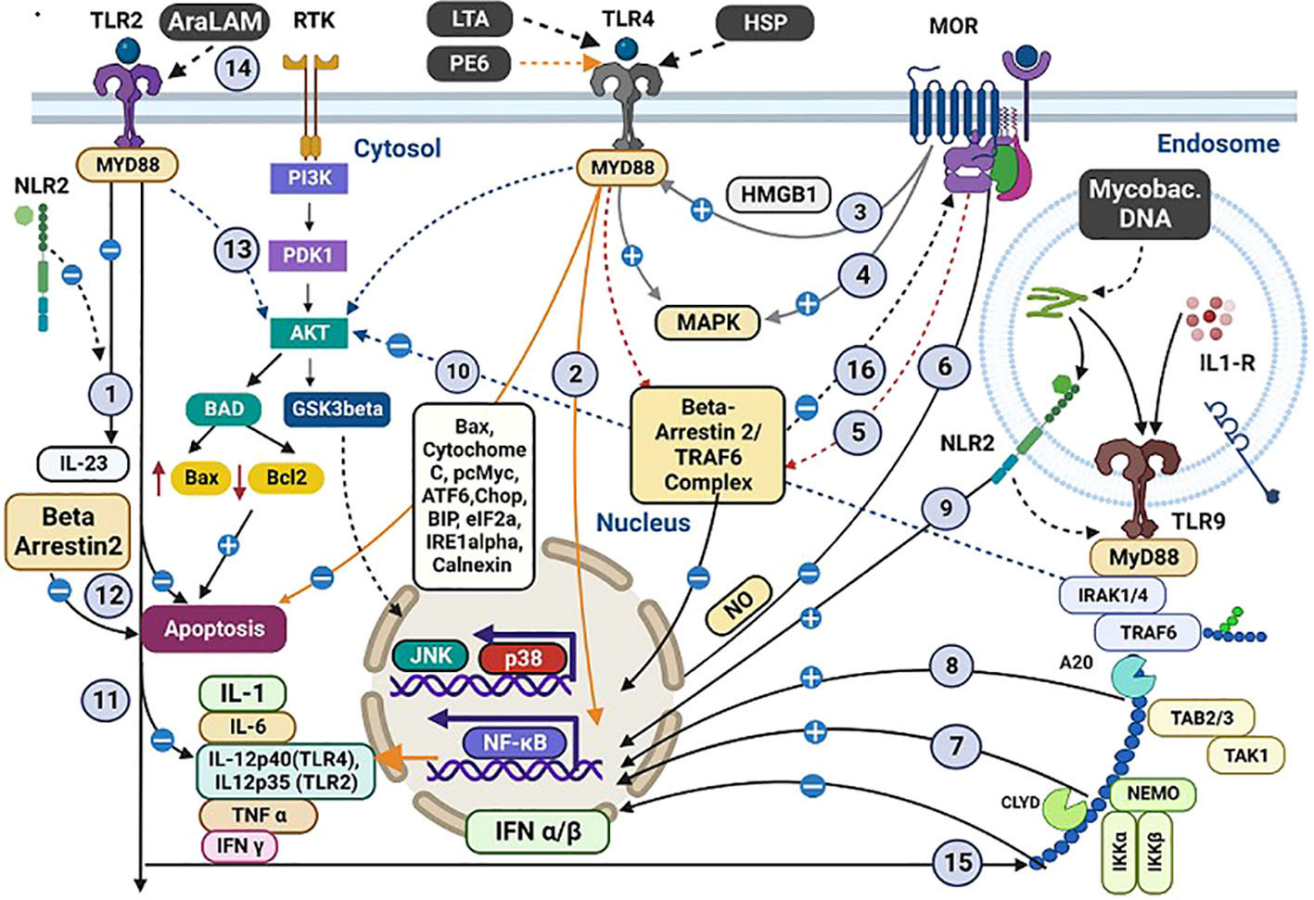
Co-stimulation of OR, TLR, and NLR with mycobacterial antigens and morphine and their intracellular cross-talking. Interactions between Mycobacterial antigens, morphine (M), and various receptors within macrophages. Both M and Mycobacterial antigens engage TLR 2/4/9. Specific antigens (LTA, HSP, PE6) interact with TLR4, inducing proinflammatory cytokines and apoptosis. Several interactions and pathways are highlighted: (1) M and bacterial antigens induce cross-talk between TLR2 and NLR2, inhibiting IL23 synthesis. (2) PE6 triggers proinflammatory cytokines via TLR4, possibly blocked by M. (3) M indirectly induces HMGB1, promoting TLR4 upregulation. (4) TLR4/OR pathways activate MAPK with proinflammatory effects in the CNS. (5) Intracellular TLR4/OR cross-talk affects cytokine secretion. (6) M inhibits LPS-induced NFκB activity. (7) M and M. tuberculosis induce TLR9 expression with pro-inflammatory consequences. (8) TLR9 regulates mycobacteria-induced Th1 responses. (9) NLR2 activates NFκB and cross-talks with TLR9. (10) M influences apoptosis-related molecules. (11) TLR2 plays a role in cytokine production regulation. (12) β-arrestin-2 negatively regulates TLR2-mediated apoptosis. (13) TLR2 activates PI3K/Akt signalling with M. (14) AraLAM induces cell activation and MTB killing. (15) TLR2-dependent inhibition of TLR9-dependent IFN α/β expression leads to decreased MHC-I cross-processing. (16) β-arrestin-2 regulates GPCR desensitization. These interactions reveal intricate immune responses in tuberculosis and opioid co-stimulation. (*A more detailed version of this figure legend is provided in the **Online Data Supplement***).

Morphine interacts with TLR2, TLR4, and CD14 cells, causing inhibitory effects. (**Figures 2**, **3**) These effects of morphine are exerted on different immune cell types interrupting their functions, which are vital for the immune defence against TB. Further, endomorphin-1, the endogenous form of opioids, has been shown to down-regulate TLR expression as a part of the negative feedback control. Consequently, external opioids, when strongly influencing the same pathway, may contribute to impaired and delayed antigen processing. (114) Consequently, the cells’ capacity to interact with mycobacterial antigens and trigger a protective immune response is ultimately reduced.

## Cross-talk between receptors

**Figure 3** summarises the cross-talk between OR, TLR, and nucleotide-binding and oligomerisation domain-like receptors (NLR) 2 via multiple cytosolic second messengers during the interaction with morphine and mycobacterial antigens. In the central nervous system, cross-taking between OR and TLR share common cytosolic molecules such as MAPK, β-arrestin-2/TRAF6 complex, and the DNA-binding protein HMGB1. (115) NLR2 on the immune cells interacts directly with mycobacterial antigens and cross-talks with TLR 2/9 to modulate the immune response. (61) Cross-talk between MOR and TLR in cancer models has shown decreased NK cytotoxicity, decreased leucocyte migration, suppression of mast cell recruitment, and the induction of M2 cell polarisation, which may contribute to the immune impairment in TB (116). Further exploration of the role of these compounds within the immune system in modulating cellular function is required.

## Variations in the antigenic stimulation

Co-stimulation of TLRs with antigenic material of a pathogen, together with morphine, has shown entirely different effects than the binding of either alone. Extensive *in vitro* and *in vivo* studies have consistently shown that morphine binding to TLR4 triggers a proinflammatory cytokine response downstream. In contrast, its binding to OR (opioid receptors) elicits an anti-inflammatory response. (104, 117) We hypothesise that opioid compounds interact with TLR as other natural compounds, modulating the host immune response, and it needs direct testing of this in pre-clinical models in the presence of tuberculosis antigens. (**Figure 3**).

Morphine causes an antiinflammatory response in dendritic cells (DC) cells via TLR2 and NLR2 when co-stimulated with *S. pneumoniae*, in contrast to the proinflammatory response induced by *S. pneumoniae* alone (**Figure 3**). (49) Morphine has also been shown to inhibit the TLR9 pathway when co-binding with HIV, promoting its replication in macrophages. (105) Similar variations of the immune effects have been observed with morphine and mycobacterial antigens in preclinical studies. Plasmacytoid DC expresses TLR9 in both humans and mice. (118–120) *M. tuberculosis* and morphine cotreatment have significantly upregulated TLR9 expression in mice. Its role is more proinflammatory, enhancing the levels of critical cytokines including TNF α, (interleukin) IL1β, and IL6, which contrasts with the antiinflammatory response exerted by TLR2/4 when co-stimulated by the same. (20, 121) This contracting proinflammatory action of TLR9 compared to other TLRs indicates the downstream receptor action heterogeneity, possibly explained by the unique binding of TB antigens with TLR9.

## Discussion

### Immunomodulatory effects of morphine in the immunopathogenesis of tuberculosis

**Figure 4:**
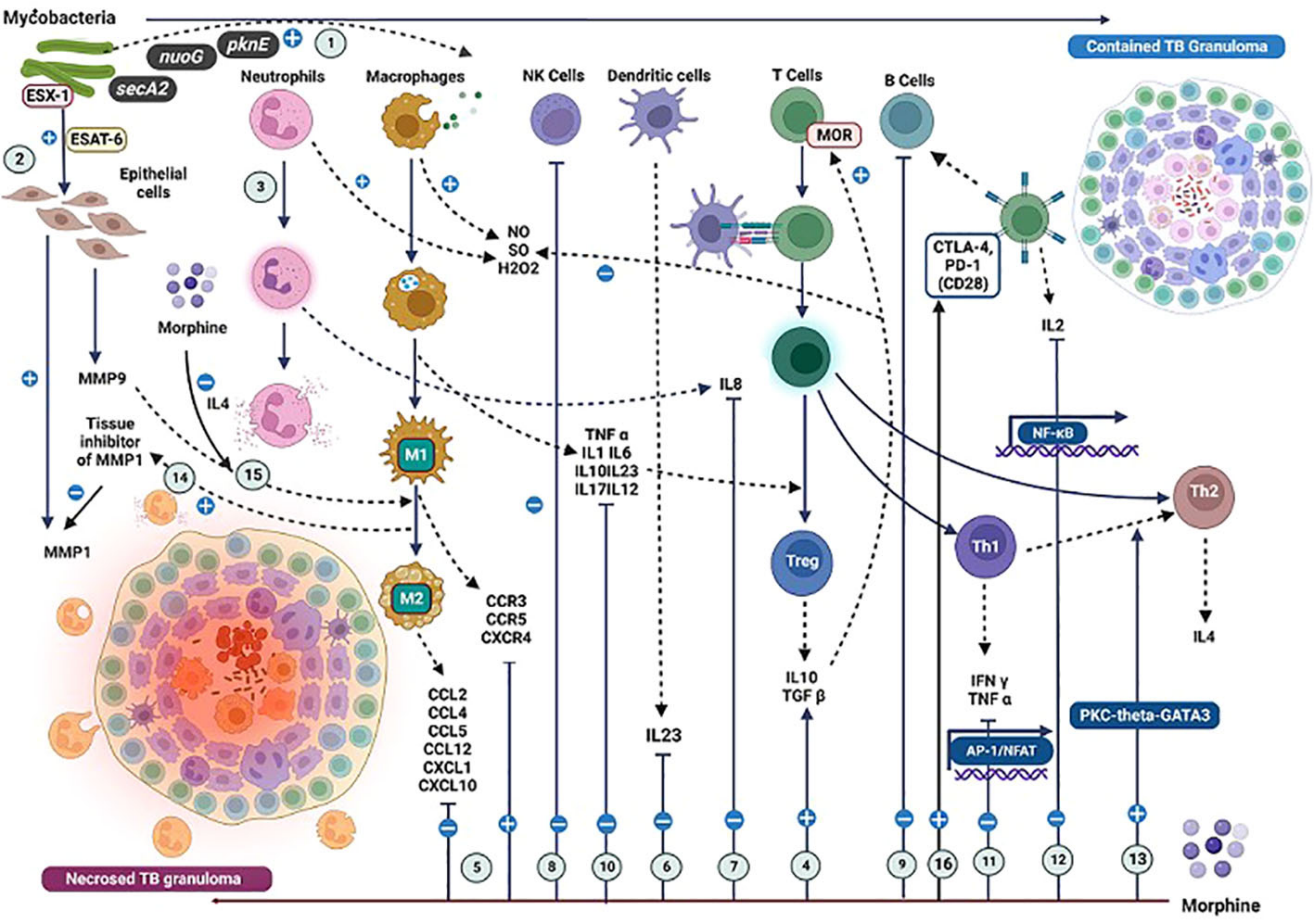
Effects due to the interactions of mycobacterial antigens and morphine with the immune cells and their mediators in the context of TB immunopathogenesis. The impact of mycobacterial antigens and morphine (M) on immune responses in tuberculosis (TB) immunopathogenesis. (1) TB bacilli virulence factors trigger a robust immune response. (2) ESX-1 induces MMP9 and MMP1 secretion; M reduces MMP9 and elevates MMP1 levels. (3) Neutrophils reduce initial Mycobacterial load. (4) M enhances TGFβ, lowering reactive oxygen intermediates. (5) M modulates CCR and CCL levels, affecting IFNγ-positive CD4 T cell migration. (6) M inhibits IL23 by dendritic cells. (7) M inhibits IL8 from neutrophils. (8) M suppresses NK cells. (9) M suppresses B cell activity. (10) M inhibits IL1, TNFα, and IL12, impacting IFNγ-induced T cell responses. (11) M inhibits IFNγ and TNFα from Th1 cells. (12) M inhibits IL2 from T cells. (13) M induces Th2 cell differentiation. (14) M promotes M2 macrophage switch via IL4. (15) MMP9 inhibition leads to M2 macrophage transformation. (16) Methadone increases CTLA-4 expression with variable PD-1 expression, impacting immune regulation in TB. (*A more detailed version of this figure legend is provided in the **Online Data Supplement***).

In the immunopathogenesis of tuberculosis, morphine exerts immunomodulatory effects, as depicted in **Figure 4**. The acquisition of TB bacilli occurs through the inhalation of respiratory droplets containing the organism. Morphine’s influence leads to the suppression of NK cells and DC, as highlighted in **Table 1**. Consequently, this suppression can impair the initial defence against TB bacilli, including nonspecific killing and antigen presentation by these cells, ultimately increasing the host’s susceptibility to TB infection.

## Effects on the TB granuloma

A TB granuloma is a unique pathological entity comprising activated macrophages, monocytes, DC, neutrophils, and T lymphocytes (**Figure 4**). An established granuloma contains infected activated macrophages and epithelioid cells forming a central necrotic core and activated macrophages and layers of CD4+ and CD8+ T cells defining a dense cellular wall encircling the necrotic core. (122, 123) It is a dynamic structure that controls two processes: the induction of apoptosis of infected macrophages and the recruitment of uninfected macrophages by creating a chemotactic gradient (123).

Granuloma formation is triggered by the mycobacterial virulence factor ESX-1 (124) It triggers matrix metallopeptidase (MMP)9 secretion by the epithelial cell matrix surrounding a granuloma (**Figure 4**). (125) It potentially induces the chemotaxis of macrophages via (chemokine ligand (CCL)7, a substrate for MMP9 produced by macrophages. (126–129) Both well-coordinated processes of new macrophage recruitment and infected macrophage apoptosis are essential to maintain the immune integrity of TB granulomas. Morphine decreases the levels of MMP9 and increases the tissue inhibitor of MMP1, dysregulating this process (**Figure 4**) (108).

### Macrophages

Macrophages, crucial in granuloma formation, face inhibition through various mechanisms induced by morphine (**Figure 2**). When infected with M. tuberculosis, macrophages experience heightened caspase-8-dependent apoptosis due to TLR2 signalling. However, mycobacteria take advantage of this situation during the initial stages of infection, as they depend on macrophages to penetrate deeper tissues and subsequently undergo apoptosis to expand within the granuloma. (123, 130) Morphine causes enhancement of TLR9-induced apoptosis of macrophages by stimulating TLR9 signalling, and multiple chemical mediators also potentiate apoptosis (**Figures 1**, **2**). (20, 59) The impact of the induction of apoptosis by morphine depends on the exact stage of the infection.

MOR located on macrophages inhibits chemotaxis, which supports the notion of an antiinflammatory role of MOR. (131) Morphine’s effects on phagocytosis were variable, with inhibition observed through a naloxone-reversible mechanism. Mycobacteria employ the cell wall-associated lipid Phthiocerol dimycocerosate (PDIM) to conceal underlying pathogen-associated molecular patterns (PAMPs), effectively evading the recruitment of microbicidal macrophages via TLR-dependent pathways. Additionally, a structurally related molecule called Surface-associated Phenolic glycolipid induces the expression of CCL2, leading to the recruitment and infection of CCR2-expressing macrophages. Morphine suppresses CCL2, counteracting this pathway and suppresses multiple chemokines important in chemotaxis (**Table 3**).

### Lymphocytes

Studies have shown that the reduction in total T cell counts and altered CD4/CD8 cell ratios are caused by morphine. Suppression of IL2 levels by morphine leads to a drop in T cell count (**Table 2**). CTLA-4 and PD-1 are two members of the CD28 family of receptors involved in T-cell inhibition by morphine. (132–134) In murine studies, MOR agonists have been shown to upregulate the expression of MOR, DOR, CD28, CTLA-4, and PD-1, which suppresses T-cell response. However, chronic opioid use has led to increased expression of CTLA-4, with unchanged PD-1 expression favouring an anti-inflammatory response among humans. (135) Morphine further triggers the Th2 switch, which may impair the cytotoxic potential of T cells against TB bacilli (**Figure 4**). Further studies are required to explore variable immune effects caused by different opioids on T cells.

### Neutrophils

Neutrophils are abundant in both early granulomas and late cavitary granulomas. (32, 136) They exhibit reduced NADPH oxidase-dependent mycobacterial killing when they ingest mycobacteria. However, their role in mediating the clearance of infected, dying macrophages appears to be host-protective. This mechanism lowers the mycobacterial load and reduces intercellular spread into uninfected macrophages. (123) The inhibition of neutrophils and IL8 secretion by morphine may lead to reduced neutrophil-mediated killing, increasing the risk of TB bacilli dissemination (**Table 3**).

### Interferons

IL17 recruits Th1 cells that secrete antigen-specific IFN γ, inhibiting MTB growth. Th1-mediated IFN γ is the critical chemical controller in granuloma formation. *M. tuberculosis* induces an IFN γ response through TLR9’s action. (21) Once stimulated by TB antigens via TLRs, macrophages and DCs secrete cytokines, including IL-12 and IL23, to induce IFN-γ production by T and NK cells. IFN-γ increases phagocytosis, phagolysosomal fusion, oxidative burst, and other nonoxidative mechanisms. (137) For an effective T helper 1 (Th1, IFN-γ producer cells) differentiation, costimulation (e.g., CD40L-CD40 and CD28-CD80/CD86 interactions) and NF-κB dependent signalling are essential. (138) IFN γ deficiency leads to a failure in granuloma formation, with subsequent infiltration of neutrophils leading to cellular necrosis. (122, 123) Bloom et al. have shown that macrophage-induced NO is the primary bactericidal mechanism of macrophages. It is established that IFN γ is an inducer of macrophage inducible NO synthase that leads to the production of NO (**Figure 2**). (139) IL1β is another mediator induced by mycobacterial antigens, which upregulate iNOS and subsequent NO production. NO-mediated killing by macrophages is the primary mechanism for controlling mycobacterial replication. A hypothesis can be proposed that the inhibitory effects of morphine on IFN γ, NO, and IL1β may lead to a dysregulation of this process, ultimately exerting negative impacts on granuloma formation (**Figure 4**; **Table 2**). Therefore, the suppression of INF-γ by morphine induces multiple significant negative implications on the immune defence against TB.

The role of IFN α/β on TB immunity is highly variable in contrast to the protective role of IFN-γ. Type I IFNs (IFN α/β) are potent inhibitors of IL-12 production by macrophages, which induces IFN-γ. (140) Conversely, they induce IFN-γ production by T and NK cells in an IL-12-independent way. (141) IFNα/β is shown to reduce monocyte viability. compromises their bacteriostatic activity and antigen presentation ability. (142) Type I IFNs have been used as an adjunctive therapeutic agent for PTB patients harbouring multi-drug resistant MTB strains. (143, 144)Multiple studies have reported that the induction of Type I IFNs precedes the onset of clinical tuberculosis. (145, 146) MTB inhibits the production of IFN α/β in response to TLR9 signalling. Morphine further suppresses this, producing complex effects requiring further characterisation in controlled studies (**Table 2**; **Figure 3**).

## Tumour necrosis factors

*M. tuberculosis*–induced TNF α production appears to be controlled via TLR2. (122) Both TNF α deficiency and excess can lead to granuloma necrosis. (147, 148) The TNF α signalling deficiency in mice produced disorganised tuberculous granulomas. (149, 150) Deficient TNF α signalling increases intra-macrophage mycobacterial load and accelerates the formation of disorganised granulomas, ultimately leading to granuloma necrosis (130, 151). Morphine has been shown to suppress TNFα levels in many studies, which may enhance the progression locally (**Tables 2**, **3**). It may further affect disorganised secondary granuloma formation in distal organs, leading to disseminated disease.

## Interleukins and chemokines

Morphine upregulates CCR expression while downregulating CCL levels, causing a net deficiency of CCL. (**Figure 2**) (21) IL6 stimulates macrophage and cytotoxic T-cell differentiation. At the same time, IL10 inhibits proinflammatory cytokines, blocks the generation of ROI and NOI, blocks antigen processing and presentation in different APCs, and diminishes T-cell responses. IL12 is a crucial cytokine in developing and maintaining type 1 cellular response in MTB infection. IL12 binds to its receptor IL12R-β2 and activates the JAK-STAT pathway, inducing IFN γ to differentiate CD4+ T cells into Th1 effectors. Preclinical evidence has shown that IL-12 p40−/− deficient mice could not control bacterial growth, which appeared to be linked to the absence of both innate and acquired sources of IFN-γ. (152) This shows the central role played by IL12 in the defence against TB infection. IL23 induces IL17 production by memory T cells, creating an inflammatory response by Th17 cells, and it generates protective cellular responses. Morphine blocks the synthesis of all these vital mediators and damages the chemical coordination in the immune defence against TB. Moreover, IL12 induces inflammation by suppressing TGF ß and stimulating NK cells, contributing to increased CCL2 and CCL3 levels. (153) However, this effect may be counteracted by overall anti-inflammatory actions caused by morphine and the virulence mechanisms of mycobacterial antigens. (**Table 3**, **Figure 4**).

## Conclusion

Chronic morphine administration causes suppression of multiple protective immune pathways vital in the defence against MTB. Multiple cellular receptors in immune cells, including OR, TLR2, and NLR 2, play critical roles in immunosuppression via complex intracellular cross-talk. Various cell types and their mediators involved in granuloma formation are inhibited by morphine via multiple mechanisms. This leads to a state of immunodeficiency that likely contributes to the reactivation, progression, and dissemination of MTB. Further studies are required to characterise potential therapeutic immunomodulatory targets in chronic opioid users at risk of infection with/reactivation of MTB.

## Limitations

*In vitro* and *in vivo* preclinical studies assessing the immunomodulatory properties of opioids have been mostly limited to the testing of morphine. Considering the wide structural diversity and functional variation of opioids, direct testing of other categories of opioids and their antagonists is needed to delineate further the postulated mechanisms of the immunopathogenesis of TB potentiated by chronic opioid use. The duration and dosage of morphine use in patients may vary widely, with the added effect of the landscape of genetic heterogeneity across cultures, the impact of this review may be biased towards findings reported from the majority of studies originating from the Western, further studies are desperately needed from third-world nations investigating this phenomenon associating opioid use and the predisposition to TB. Chronic opioid use is associated with confounding factors, socioeconomic deprivation, malnutrition and infections associated with IVDU which may contribute to immune suppression.

## Data availability statement

The original contributions presented in the study are included in the article/**Supplementary Material**. Further inquiries can be directed to the corresponding author.

## Author contributions

VB: Conceptualization, Data curation, Methodology, Software, Visualization, Writing – original draft. HS: Data curation, Formal Analysis, Writing – review & editing. NR: Supervision, Writing – review & editing. LD: Supervision, Writing – review & editing.
