# Supplementary material for: Immunomodulatory properties of morphine and the hypothesised role of long-term opioid use in the immunopathogenesis of tuberculosis
Source: Front Immunol. 2023 Oct 23;14:1265511. doi: 10.3389/fimmu.2023.1265511 (PMC10628761; doi:10.3389/fimmu.2023.1265511)
Supplement: Supplementary file 1 [file DataSheet_1.docx]

**Supplementary detailed figure legends**

**Figure 2 illustrates the interaction between tuberculous antigens and morphine with a macrophage's cell surface and intracellular receptors.** *(Morphine molecules are shown as black dots, and the Mycobacterial antigens are shown as black rectangles.)* Both morphine (M) and Mycobacterial antigens interact with TLR 2/4/9. Mycobacterial antigens interact with NLR, MR, and the Dectin-1 receptor (TLR9 is located on the endosomes and interacts with mycobacterial DNA) and M with OR. M adds to the increased virulence of mycobacterial antigens due to the inhibitory effects exerted via TLR 2/4 and OR. (1) M cross desensitises multiple CCR via MOR and DOR.(1) (2) M inhibits FcR-mediated apoptosis via MOR, DOR, and KOR.(2,3) (3) M enhances TGF-induced apoptosis. (4) (4) M inhibits the synthesis of NO and SO, impairs respiratory burst activity and the killing of bacilli. (5) NLR2 potentiates the actions of TLR 2/4 via cross-talking. (6) M inhibits NF kB-mediated cytokine and chemokine synthesis by cross-talking between OR and TLR. TLR9 induced a proinflammatory response with Mycobacterial antigens and M, while TLR2/4 caused an anti-inflammatory response.(5,6) (7) MR is present only in AM. *MTB*or ManLAM upregulates PPARγ via MR, increasing IL8, COX_2,_ and PGE_2_. Overall, MR negatively regulates protective macrophage inflammatory responses against *MTB*.(7) (8) ManLAM generates an antiinflammatory response by inhibiting proinflammatory TNF and IL12 and inducing immunosuppressive IL10 and TGF β. (9) ManLAM signalling through TLR2 and TLR4 in monocytes triggers chemokine secretion. M has inhibitory effects via TLR 2/4. (10) Dectin-1, a β-glucan receptor, in combination with TLR2, induces TNF production in macrophages, particularly those infected with attenuated MTB strains. (11) LM blocks TLR2-induced biosynthesis of TNF in macrophages, thereby allowing MTB to subvert the host immune response. Antigen-specific variations are observed, and the immune suppression caused by M further potentiates antigen virulence mechanisms.(7) [CCR, Chemokine receptor; COX2, Cyclooxygenase 2; CXCL, C-X-C motif chemokine ligand; CXCR, C-X-C Motif chemokine receptor; DOR, δ-opioid receptor; FcR, Fc Receptor; IL, Interleukins; KOR, κ-opioid receptor; LM, Lipomannan; MANLAM, Mannosylated lipoarabinomannan; MOR, μ-opioid receptor; MR, Mannose receptor; NFκB, Nuclear factor kappa B; NLR, Nucleotide oligomerisation domain-like receptor; NO, Nitrous oxide; PGE2, Prostaglandin E2; PPAR-γ, Peroxisome proliferator-activated receptor gamma; SO, Sulphur oxide; TGF, Transferrin growth factor; TLR, Toll-like receptor; TNF, Tumour necrosis factor: ***Plus sign:*** *stimulation;* ***minus sign:*** *inhibition*].

**Figure 3: Illustration of OR, TLR, and NLR co-stimulation with Mycobacterial antigens and morphine and their intracellular cross-talking.** *[Some examples of cotreatment with morphine and bacterial LPS and selected opioid interaction pathways in the brain are included in the illustration for comparison and to build up the hypothesis]*. Mycobacterial antigens and morphine (M) interact with TLR 2/4/9.(8–10) LTA, HSP, and PE6 are mycobacterial antigens that interact with TLR4 to promote proinflammatory cytokines, which induce apoptosis. (9,11) (1) Co-treatment of bacterial antigens (S. pneumoniae) and M induces cross-talk between TLR2 and NLR2, leading to the inhibition of IL23 synthesis via the TLR2-MyD88-IRAK1/4 pathway (8) (2) PE6 induces the secretion of proinflammatory cytokines via activation of NF kB through TLR4 with adaptor Myd88, blocked by M (orange arrow). PE6 strongly induces apoptosis via increased production of pro-apoptotic molecules shown. These effects are possibly inhibited by M. (9,12) (3) M induces the production of HMGB1 (indirectly), an endogenous TLR4 agonist that supports intercellular (neuron-to-glia or glia-to-neuron) interactions in the brain. HMB1 promotes TLR upregulation. (4) TLR4 and OR pathways activate MAPK, and this cross-talk produces proinflammatory effects in the CNS, affecting p38 and JNK promoters. This contrasts with the immune cells, wherein the effect is anti-inflammatory. (5) The intracellular TLR4/OR signalling pathway cross-talking induces the β-arrestin-2/ TRAF6 complex, contributing to M-induced inhibition of LPS-induced TNF-α secretion in mast cells.(13) (6). M inhibits LPS-induced NF kB nuclear binding in human blood neutrophils and monocytes in a time-, concentration- and naloxone-sensitive-dependent manner.(14) (7) The combination of M and H37Ra infection in WT mice has a pro-inflammatory role, and it induces TLR9 expression that results in inflammation. TLR9 is an important factor in the host’s resistance to opioids and *M. tuberculosis* infection. (15) (8) M and *M. tuberculosis* significantly induce the expression of TLR9 in both DC and macrophages and enhance the levels of cytokines in WT mice. *M. tuberculosis* DNA act via TLR9 and induce the expression of IL12p40, TNF, IFN α, and TNF, IL6 by DC and macrophages, respectively. TLR9 plays an important role in the regulation of mycobacteria-induced Th1 responses. (7,15) (9) NLR2 interacts with mycobacterial antigens to activate downstream NF κB, promoting inflammatory mediators and cross-talks with TLR9 to potentiate the same effects.(16) (10) M and *M. tuberculosis* R37Ra (attenuated *Mycobacterium tuberculosis* strain) and TLR9 agonist CpG-DNA induce the phosphorylation of BAD, a pro-apoptotic member, through the activation of Akt. Phosphorylated BAD expresses antiapoptotic effects. The treatment with M of an R37Ra infection decreases the levels of Bcl-2 and increases the levels of Bax. The Bcl-2 family is involved in TLR9-mediated apoptosis in the lung following morphine and H37Ra administration. Mice deficient in TLR9 and TLR2 show enhanced susceptibility to *M. tuberculosis*, proving their additive effects.(15,17) (11) AraLAM from rapidly growing non-TB mycobacteria activates murine macrophages via TLR2, in which M. TLR2 plays an important role in regulating mycobacterial-induced cytokine production. TLR2/9−/− mice have displayed a profound reduction in IL12p40.(17,18) (12) β-arrestin-2 is a negative regulator in M-induced, TLR2-mediated apoptosis. In TLR signalling, β-arrestin interacts with TRAF6 and negatively regulates the downstream signalling pathways.(19) (13) TLR2 activates the PI3K/Akt signalling pathway with M. Activated Akt phosphorylate GSK3β, a constitutively active enzyme, is inactivated by Akt. (15) (14) AraLAM was shown to induce cell activation via TLR2, leading to MTB killing in both murine and human macrophages in a NO-dependent and independent manner, respectively.(7) (15) The TLR2-dependent inhibition of TLR9-dependent IFN α/β expression leads to a decrease in IFN-α/β-dependent MHC-I cross-processing, which is an anti-inflammatory action of TB.(7) (16) β-arrestin-2 regulates the desensitisation of GPCR. [AraLAM, abinofuranosyl-capped lipoarabinomannan; BAD, Bcl2 associated agonist of cell death; Bax, Bcl-2-associated X protein; Bcl-2, B-cell lymphoma-2; CNS, Central nervous system; DC, Dendritic cells; DNA, Deoxyribonucleic acid; GPCR, G Protein-coupled receptor; GSK3β, Glycogen synthase kinase 3-beta; HMGB1, High mobility group box protein 1; HSP, Heat shock proteins; JNK, c-Jun N-Terminal kinase; LPS, Lipopolysaccharides; LTA, Lipoteichoic acid; MAPK, Mitogen-activated protein kinase; MHC, Major histocompatibility complex; MTB, Mycobacterial tuberculosis; MyD88, Myeloid differentiation primary response 88; NFκB; Nuclear factor kappa B; NLR, Nucleotide oligomerisation domain-like receptor; NO, Nitrous oxide; OR, Opioid receptor; PE6, Proline–glutamic acid 6; PI3K, Phosphoinositide 3-kinase; TB, Tuberculosis; TLR, Toll-like receptor; TNF, Tumour necrosis factor; TRAF6, TNF receptor-associated factor 6; WT, Wild type: ***Plus, sign:*** *stimulation;* ***minus sign:*** *inhibition*].

**Figure 4: Illustration of the effects due to the interactions of mycobacterial antigens and morphine with the immune cells and their mediators in the context of TB immunopathogenesis.** (1) The virulence factors of TB bacilli (NuoG, SecV2, and PknE) trigger a strong immune response. (2) ESX-1 induces epithelial cells to secrete MMP9 via its protein substrate ESAT-6, and MMP1 is secreted (20).(21) Morphine (M) decreases the serum levels of MMP9 and increases the levels of the tissue inhibitor of MMP1. (3) Neutrophils are the predominant phagocytic cells in the airways in active pulmonary TB and help to reduce initial *Mycobacterial* load. (22) (4) M potentiates TGF β production by lymphocytes, which mediate the M-induced downregulation of reactive oxygen intermediates (NO, H_2_O_2,_ and SO). TGF β induces significant increases in MOR transcript expression in T cells. (22–25) (5) M upregulates CCR expression while downregulating CCL levels. CCL5 and CCL4 and their receptors directly control the migration of IFN γ-positive CD4 T cells to the site of the infection in the formation of early granulomas in mice. (26,27) (6) M inhibits IL23 produced by DC. (7) M inhibits IL8 production by neutrophils. (8) M inhibits NK cells directly and by a centralised mechanism. (9) M suppresses B cell activity. (10) M suppresses IL1, TNF α and IL12, and the effects of IL10 synthesis are variable. The inhibition of IL12 leads to reduced levels of IL23 and subsequent low IFN γ-induced T cell responses in subjects susceptible to Mycobacterial infections. (27–29) (11) M inhibits the synthesis of IFN γ and TNF α from Th1 cells via AP/NTAT. (12) M inhibits the synthesis of IL2 from T cells, acting via NF kB. (13) M induces the differentiation of T helper cells to Th2 effector cells via PKC-θ-GATA3.(30) (14) M increases M2 switch, and the resulting IL4 induces the inhibition of MMP9. (15) The inhibition of MMP9 induces the transformation of M2 macrophages. (16) Opioids (methadone) increase the expression of CTLA-4 with variable PD-1 expression.(31) [CCL, Chemokine ligand; CCR, Chemokine receptor; CTLA-4, Cytotoxic T lymphocyte-associated antigen-4; DC, Dendritic cells; ESAT-6, Early secreted antigenic target 6; ESX-1, Early secreted antigen-6 kilodaltons (ESAT-6) system 1; H2O2, Hydrogen peroxide; IFN-γ, Interferon-gamma; IL, Interleukins; MMP, Matrix metallopeptidase; MOR, μ-opioid receptor; NK, Neutral killer cells; NO, Nitrous oxide; PD1, Programmed cell death protein 1; PKC, Protein kinase C; PKC-θ-GATA3; Protein kinase C-theta- GATA binding protein 3; SO, Sulphur oxide; TB, Tuberculosis; TGF, Transferrin growth factor; TNF, Tumour necrosis factor: ***Plus sign****: stimulation;* ***minus sign****: inhibition*].

Reference for Figure Legends 2, 3 and 4 (Mendeley App is used)

1. Grimm MC, Ben-Baruch A, Taub DD, Howard OMZ, Resau JH, Wang JM, Ali H, Richardson R, Snyderman R, Oppenheim JJ. Opiates transdeactivate chemokine receptors: delta and mu opiate receptor-mediated heterologous desensitization. *J Exp Med* (1998) 188:317–325. doi: 10.1084/JEM.188.2.317

2. Szabo I, Rojavin M, Bussiere JL, Eisenstein TK, Adler MW, Rogers TJ. Suppression of peritoneal macrophage phagocytosis of Candida albicans by opioids. *Journal of Pharmacology and Experimental Therapeutics* (1993) 267:

3. Tomassini N, Renaud F, Roy S, Loh HH. Morphine inhibits Fc-mediated phagocytosis through mu and delta opioid receptors. *J Neuroimmunol* (2004) 147:131–133. doi: 10.1016/J.JNEUROIM.2003.10.028

4. Singhal PC, Kapasi AA, Franki N, Reddy K. Morphine-induced macrophage apoptosis: the role of transforming growth factor-beta. *Immunology* (2000) 100:57–62. doi: 10.1046/J.1365-2567.2000.00007.X

5. Wang J, Barke RA, Charboneau R, Schwendener R, Roy S. Morphine induces defects in early response of alveolar macrophages to Streptococcus pneumoniae by modulating TLR9-NF-kappa B signaling. *J Immunol* (2008) 180:3594–3600. doi: 10.4049/JIMMUNOL.180.5.3594

6. Sacerdote P. Effects of in vitro and in vivo opioids on the production of IL-12 and IL-10 by murine macrophages. *Ann N Y Acad Sci* (2003) 992:129–140. doi: 10.1111/J.1749-6632.2003.TB03144.X

7. Sasindran SJ, Torrelles JB. Mycobacterium Tuberculosis Infection and Inflammation: what is Beneficial for the Host and for the Bacterium? *Front Microbiol* (2011) 2: doi: 10.3389/FMICB.2011.00002

8. Wang J, Ma J, Charboneau R, Barke R, Roy S. Morphine inhibits murine dendritic cell IL-23 production by modulating Toll-like receptor 2 and Nod2 signaling. *J Biol Chem* (2011) 286:10225–10232. doi: 10.1074/JBC.M110.188680

9. Sharma N, Shariq M, Quadir N, Singh J, Sheikh JA, Hasnain SE, Ehtesham NZ, Leigh Sampson S, and HS. Mycobacterium tuberculosis Protein PE6 (Rv0335c), a Novel TLR4 Agonist, Evokes an Inflammatory Response and Modulates the Cell Death Pathways in Macrophages to Enhance Intracellular Survival. *Article* (2021) 12:1. doi: 10.3389/fimmu.2021.696491

10. Rehli M. Of mice and men: species variations of Toll-like receptor expression. *Trends Immunol* (2002) 23:375–378. doi: 10.1016/S1471-4906(02)02259-7

11. Human Toll-Like Receptors Mediate Cellular Activation by Mycobacterium tuberculosis | The Journal of Immunology. https://www.jimmunol.org/content/163/7/3920.long [Accessed December 19, 2021]

12. McCoy CE, O’Neill LAJ. The role of toll-like receptors in macrophages. *Front Biosci* (2008) 13:62–70. doi: 10.2741/2660

13. Zhang P, Yang M, Chen C, Liu L, Wei X, Zeng S. Toll-Like Receptor 4 (TLR4)/Opioid Receptor Pathway Crosstalk and Impact on Opioid Analgesia, Immune Function, and Gastrointestinal Motility. *Front Immunol* (2020) 11:1455. doi: 10.3389/FIMMU.2020.01455/BIBTEX

14. Welters ID, Menzebach A, Goumon Y, Cadet P, Menges T, Hughes TK, Hempelmann G, Stefano GB. Morphine inhibits NF-kappaB nuclear binding in human neutrophils and monocytes by a nitric oxide-dependent mechanism. *Anesthesiology* (2000) 92:1677–1684. doi: 10.1097/00000542-200006000-00027

15. Chen L, Shi W, Li H, Sun X, Fan X, LeSage G, Li H, Li Y, Zhang Y, Zhang X, et al. Critical Role of Toll-Like Receptor 9 in Morphine and Mycobacterium tuberculosis–Induced Apoptosis in Mice. *PLoS One* (2010) 5:e9205. doi: 10.1371/JOURNAL.PONE.0009205

16. Chen G, Shaw MH, Kim YG, Nuñez G. NOD-Like Receptors: Role in Innate Immunity and Inflammatory Disease. *https://doi.org/101146/annurev.pathol4110807092239* (2009) 4:365–398. doi: 10.1146/ANNUREV.PATHOL.4.110807.092239

17. Bafica A, Scanga CA, Feng CG, Leifer C, Cheever A, Sher A. TLR9 regulates Th1 responses and cooperates with TLR2 in mediating optimal resistance to Mycobacterium tuberculosis. *J Exp Med* (2005) 202:1715. doi: 10.1084/JEM.20051782

18. Yoshimura DT, Golenbock MJ, Fenton TK, Means S, Wang E, Lien A. Mycobacterium tuberculosis Activation by Human Toll-Like Receptors Mediate Cellular. *J Immunol References* (1999) 3920:3920–3927. http://www.jimmunol.org/content/163/7/http://www.jimmunol.org/content/163/7/3920.full#ref-list-1 [Accessed December 19, 2021]

19. Li Y, Sun XL, Zhang Y, Huang JJ, Hanley G, Ferslew KE, Peng Y, Yin DL. Morphine promotes apoptosis via TLR2, and this is negatively regulated by beta-arrestin 2. *Biochem Biophys Res Commun* (2009) 378:857–861. doi: 10.1016/J.BBRC.2008.12.001

20. McDonald J, Lambert DG. Opioid receptors. *Continuing Education in Anaesthesia Critical Care & Pain* (2005) 5:22–25. doi: 10.1093/BJACEACCP/MKI004

21. Pagán AJ, Ramakrishnan L. Immunity and Immunopathology in the Tuberculous Granuloma. *Cold Spring Harb Perspect Med* (2015) 5: doi: 10.1101/CSHPERSPECT.A018499

22. Eum SY, Kong JH, Hong MS, Lee YJ, Kim JH, Hwang SH, Cho SN, Via LE, Barry CE. Neutrophils are the predominant infected phagocytic cells in the airways of patients with active pulmonary TB. *Chest* (2010) 137:122–128. doi: 10.1378/CHEST.09-0903

23. Rogers TJ. Bidirectional Regulation of Opioid and Chemokine Function. *Front Immunol* (2020) 11:94. doi: 10.3389/FIMMU.2020.00094

24. Al-Hashimi M, Scott SWM, Thompson JP, Lambert DG. Opioids and immune modulation: more questions than answers. *Br J Anaesth* (2013) 111:80–88. doi: 10.1093/BJA/AET153

25. Barnes PJ. Receptor heterodimerization: a new level of cross-talk. *J Clin Invest* (2006) 116:1210–1212. doi: 10.1172/JCI28535

26. Eisenstein TK. The Role of Opioid Receptors in Immune System Function. *Front Immunol* (2019) 10:2904. doi: 10.3389/FIMMU.2019.02904/BIBTEX

27. Long X, Li Y, Qiu S, Liu J, He L, Peng Y. MiR-582-5p/miR-590-5p targeted CREB1/CREB5–NF-κB signaling and caused opioid-induced immunosuppression in human monocytes. *Translational Psychiatry 2016 6:3* (2016) 6:e757–e757. doi: 10.1038/tp.2016.4

28. Roach TI, Barton CH, Chatterjee D, Blackwell JM. Macrophage activation: lipoarabinomannan from avirulent and virulent strains of Mycobacterium tuberculosis differentially induces the early genes c-fos, KC, JE, and tumor necrosis factor-alpha. *J Immunol* (1993) 150:1886–1896. https://europepmc.org/article/MED/8436823 [Accessed December 25, 2022]

29. Reinecke BA, Kang G, Zheng Y, Obeng S, Zhang H, Selley DE, An J, Zhang Y. Design and synthesis of a bivalent probe targeting the putative mu opioid receptor and chemokine receptor CXCR4 heterodimer. *RSC Med Chem* (2020) 11:125–131. doi: 10.1039/C9MD00433E

30. Han C, Lei D, Liu L, Xie S, He L, Wen S, Zhou H, Ma T, Li S. Morphine induces the differentiation of T helper cells to Th2 effector cells via the PKC-θ-GATA3 pathway. *Int Immunopharmacol* (2020) 80: doi: 10.1016/J.INTIMP.2019.106133

31. Mazahery C, Benson BL, Cruz-Lebrón A, Levine AD. Chronic Methadone Use Alters the CD8 + T Cell Phenotype In Vivo and Modulates Its Responsiveness Ex Vivo to Opioid Receptor and TCR Stimuli . *The Journal of Immunology* (2020) 204:1188–1200. doi: 10.4049/JIMMUNOL.1900862/-/DCSUPPLEMENTAL
